# The Effect of Continuous Theta Burst Stimulation over the Right Dorsolateral Prefrontal Cortex on Cognitive Function and Emotional Regulation in Patients with Cerebral Small Vessel Disease

**DOI:** 10.3390/brainsci13091309

**Published:** 2023-09-11

**Authors:** Pei Dai, Zhao-Xia Wang, Hui-Xian Yu, Chang-Bin Liu, Si-Hao Liu, Hao Zhang

**Affiliations:** 1 School of Rehabilitation, Capital Medical University, China Rehabilitation Research Center, Beijing 100068, China; 2 Department of Rehabilitation Medicine, Beijing Tian tan Hospital, Capital Medical University, Beijing 100070, China; 3 China National Clinical Research Center for Neurological Diseases, Beijing 100070, China

**Keywords:** cerebral small vessel disease, cognitive function, Hamilton depression score, continuous theta burst stimulation, right dorsolateral prefrontal cortex

## Abstract

Objectives: Cognitive impairment in cerebral small vessel disease (CSVD) is a common cause of vascular dementia and is often accompanied by mental disorders. The purpose of this study was to investigate the effect of continuous theta burst stimulation (cTBS) over the right dorsolateral prefrontal cortex (DLPFC) on the cognitive function and Hamilton depression (HAMD) scores in patients with CSVD. Methods: A total of 30 CSVD patients who met the inclusion criteria were randomly assigned to either the sham or cTBS group. The patients in both groups received routine cognitive function training. All the patients were under treatment for 14 sessions, with one session per day (each cTBS conditioning session consisted of three-pulse bursts at 50 Hz repeated at 5 Hz, 80% MT, and 600 pulses). Before and after the treatment, the patients in both groups were evaluated using the Montreal Cognitive Assessment (MoCA), Stroop Color-Word Test (SCWT), Trail Marking Test (TMT), Digital Span Test (DST), and HAMD test. The time to complete the SCWT and TMT were recorded. The scores of the MoCA, DST and HAMD test were recorded. Results: The HAMD scores in the cTBS group decreased significantly compared to the control (*p* < 0.05). There were no significant differences in the MoCA (including the MoCA subitems) or DST scores or in the SCWT or TMT completion times between the two groups (*p* > 0.05). For the HAMD scores and the MoCA subitem visuospatial/executive scores, the SCWT-B and SCWT-C completion times in the two groups both improved significantly before and after treatment (*p* < 0.05). For the MoCA scores, the DST-backward scores and the TMT-B completion times in the cTBS group improved significantly before and after treatment (*p* < 0.05). There was no significant difference in the SCWT-A, TMT-A completion times and MoCA subitems naming, attention, language, abstraction, delayed recall, and orientation scores either before or after treatment in the two groups or between the two groups (*p* > 0.05). Conclusions: In this study, cTBS over the right DLPFC decreased the HAMD scores significantly in patients with CSVD but had no significant improvement or impairment effects on cognitive function. cTBS over the right DLPFC could be used to treat CSVD patients with depression symptoms.

## 1. Introduction

Cerebral small vessel disease (CSVD) causes 25% of strokes, and the prevalence increases with age [1]. The brain imaging biomarkers of CSVD include recent small subcortical infarcts, white matter hyperintensities, lacunes, cerebral microbleeds, enlarged perivascular spaces, and cerebral atrophy [2]. CSVD is considered to be the most common cause of vessel cognitive impairment (VCI) [3], and cognitive functions such as attention, processing speed, and executive function are often involved [4]. Depression is also frequently seen in patients with CSVD. Traditional treatment methods, such as drugs to improve cognitive function and mood disorders, have some shortcomings clinically.

Repeated transcranial magnetic stimulation (rTMS) has been shown in many studies over the years to modulate cognitive function, both in animal experimentation and in patients [5,6], and to improve depression [7,8]. Theta burst stimulation (TBS) is a special mode of rTMS that includes continuous theta burst stimulation (cTBS) and intermittent theta burst stimulation (iTBS). Similar to the excitatory effect of high-frequency stimulation (>5 Hz) and the inhibitory effect of low-frequency stimulation (1 ≤ Hz) in rTMS [9], iTBS induces a long-term excitatory effect on neural function (long-term potentiation, LTP), while cTBS can rapidly induce inhibitory effects on neural function (long-term depression, LTD) [10]. In recent years, TBS has been increasingly used to improve cognitive impairment in post-stroke patients [11,12] as well as mood disorders (e.g., depression) [13]. TBS has been found to have the same cortical modulatory effect as rTMS but with fewer pulses and a higher efficiency, requiring only 20 s to 3 min to be fully effective [14,15]. The dorsolateral prefrontal cortex (DLPFC) is a crucial area for processing various behavioral tasks. However, the effects of the cTBS protocol delivered unilaterally to the right DLPFC appear to be controversial in their ability to improve cognitive function or depression [16,17,18]. Previous studies have focused more on the role of the left DLPFC since the left cerebral hemisphere is dominant in language, logical thinking, analysis, synthesis, and positive emotions [19,20]. Although the right DLPFC has also been shown to be involved in cognitive processing and negative emotions, it still seems to be ignored. In some studies, cTBS over the right DLPFC even seemed to reduce both inhibitory and cognitive control [18,21], or only exhibit a modest antidepressant effect [1].

In recent years, with the deepening of research, rapid progress has been made in the risk factors, pathogenesis, clinical manifestations, and evaluation systems of CSVD.

However, the study on the dysfunction caused by CSVD is insufficient. Cognitive impairment in CSVD has specific manifestations and is often accompanied by depressive symptoms. Few studies exist on the treatment of cognitive impairment and mental disorders with TBS in CSVD. In the context of the controversial effects of cTBS over the DLPFC, our study aimed to investigate the effects of cTBS over the right DLPFC on cognitive function and depression in patients with CSVD and to provide direction for clinical intervention.

## 2. Materials and Methods

### 2.1. Patient Selection

A total of 30 patients aged between 40 and 80 years with CSVD who were admitted to Beijing Tiantan Hospital rehabilitation department between January 2021 and April 2023 were recruited. All the patients who were enrolled provided signed and informed consent.

Inclusion criteria: ① according to the definition published by Pantoni et al. [22] in 2010, the imaging manifestations of CSVD included lacunae, new subcortical infarcts, leukoencephalopathy, an enlarged perivascular space, cerebral micro-hemorrhage, and cerebral atrophy [2]; ② a CSVD diagnosis in accordance with the criteria of the Chinese Guidelines for Diagnosis and Treatment of Cognitive Dysfunction related to Cerebral Small Vessel Disease (2019) [23]; ③ an education level of Grade 6 or above; ④ 40–80 years old; and ⑤ signed informed consent.

Exclusion criteria: ① a history of a large artery atherosclerosis stroke; ② unable to cooperate with the cognitive assessment due to language, hearing, or visual impairment; ③ other diseases affecting cognitive function (such as Alzheimer’s disease, Parkinson’s disease, epilepsy, etc.); and ④ unable to perform a head MRI due to a pacemaker, stent placement, or claustrophobia.

In our study, all the 30 CSVD patients who met the inclusion criteria were enrolled. Using the random number table method, patients were divided into two groups (*n* = 15 cases in each group). In the cTBS group, there were 11 men and four women who were aged (68.93 ± 5.09) years old and educated for (9.87 ± 2.56) years. In the sham cTBS group, there were 11 men and four women who were aged (64.93 ± 6.70) years old and educated for (9.80 ± 3.45) years. All the patients received routine cognitive function training.

### 2.2. Depression and Cognitive Function Assessment

#### 2.2.1. Primary Outcomes

##### Montreal Cognitive Assessment (MoCA)

The MoCA included visuospatial, execution, naming, memory, attention, language, abstraction, delayed recall, and orientation components. One point was added if the patient had less than 12 years of education. A score of less than 26 indicated a cognitive impairment. A higher total score indicated a better cognitive function [24].

##### Hamilton Depression Scale (HAMD)

Each participant was evaluated by a professionally trained psychiatrist using the Hamilton depression scale (HAMD). The scores were categorized as no depression (0–7), mild depression (8–16), moderate depression (17–23), and severe depression (≥24) [25].

#### 2.2.2. Secondary Outcomes

##### The Stroop Color-Word Test (SCWT)

The SCWT was used to measure the perceptual conversion ability, selective attention, and inhibitory control ability. It used three cards. Card A included 50 color words (yellow, red, blue, and green), and the subjects were asked to read out the words representing the colors as quickly and accurately as possible. Card B consisted of dots in four colors (yellow, red, blue, and green), and the subjects were asked to read out the colors as quickly and accurately as possible. Card C covered four color words, which were printed in four different colors (yellow, red, blue, and green), and the subjects were asked to read out the colors as quickly and accurately as possible. In our study, the time to complete the SCWT was recorded for all the patients. Performing the task faster was associated with a better inhibitory control of executive functioning [26].

##### The Trail Marking Test (TMT)

The TMT was divided into two parts: A and B. Part A required the subjects to connect numbers 1–25 in sequence, reflecting the function of the right cerebral hemisphere, including rapid visual search, visuospatial sorting, and perceptual motor speed ability. Part B asked the subjects to connect 25 numbers and letters alternately in sequence, reflecting the function of the left cerebral hemisphere, including overall visuospatial scanning, perceptual motion speed, and set-shifting ability [27]. The TMT completion time was recorded for all the patients. A shorter time to completion indicated a better execution function set-shifting ability.

##### The Digital Span Test (DST)

The DST contained digit forward and digit backward tests. It mainly assessed memory refresh, attention, and working memory. The evaluator read a list of numbers aloud and then asked the subject to recall the numbers either in order or in reverse order. One point was scored for each string of numbers answered correctly. The evaluator terminated the test after two consecutive failed strings, and then calculated the score. A higher score suggested better memory refresh, attention, and working memory [28].

### 2.3. Study Design and TMS Procedure

A randomized, double-blind study was designed. The patients were randomly assigned into two groups, including the cTBS group and the sham cTBS group. All the patients were blinded to the treatment. The evaluators were also unaware of the patients’ grouping.

The TMS procedures were used to apply cTBS over the right DLPFC. cTBS was performed using a figure-eight-shaped coil (each loop had a diameter of 3.5 cm) connected to a Yiruide CCY-IA magnetic stimulation device (Wuhan Yiruide Medical Equipment New Technology Co., Ltd., Wuhan, China) that was cooled in liquid nitrogen. Each cTBS conditioning session consisted of three-pulse bursts at 50 Hz repeated at 5 Hz. Thus, 600 pulses were applied at intervals of 0.02 s for a total of 40 s for each patient at an 80% threshold of the maximum output intensity.

Prior to the application of cTBS, the resting motor threshold (RMT) determination was performed. An electromyographic electrode was used to record the motor evoked potentials (MEPs) at the muscular abdomen of the first dorsal interosseous muscle. The RMT was determined as the minimum intensity required to evoke five out of 10 MEPs greater than 50 mV at rest. The cTBS group and sham groups received real cTBS or sham stimulation, respectively. In the cTBS intervention group, the center of the figure-eight-shaped coil was tangent to the intersection of the DLPFC of the skull. In the sham intervention group, the coil was held perpendicular to the skull, and the brain was not affected by the magnetic field. The parameters of the cTBS and sham groups were the same, and the stimulation protocol was performed once a day for a total of two weeks.

## 3. Statistical Analysis

GraphPad Prism 9.4.1 (GraphPad Software, Inc., San Diego, CA, USA) was used for statistical analysis and graphing. The baseline characteristics were compared between the two groups of patients. Fisher’s exact test (for percentages) was performed to evaluate the between-group sex difference. The continuous variables were expressed as the mean ± the standard deviation. The unpaired *t*-test was performed to evaluate between the groups. The non-normally distributed data were expressed as the median (M) and interquartile range (IQR). The Mann–Whitney U test was performed to evaluate the between-group differences and the Wilcoxon test was performed to evaluate the differences before and after treatment in both groups. A *p* value < 0.05 was considered statistically significant.

## 4. Results

This study of 30 patients with CSVD included 22 males and eight females. All the patients had a history of hypertension.

There were no significant differences in age, gender, or educational level (*p* > 0.05, Table 1).

### 4.1. The Effects of cTBS on the HAMD Scores

**Figure 1:**
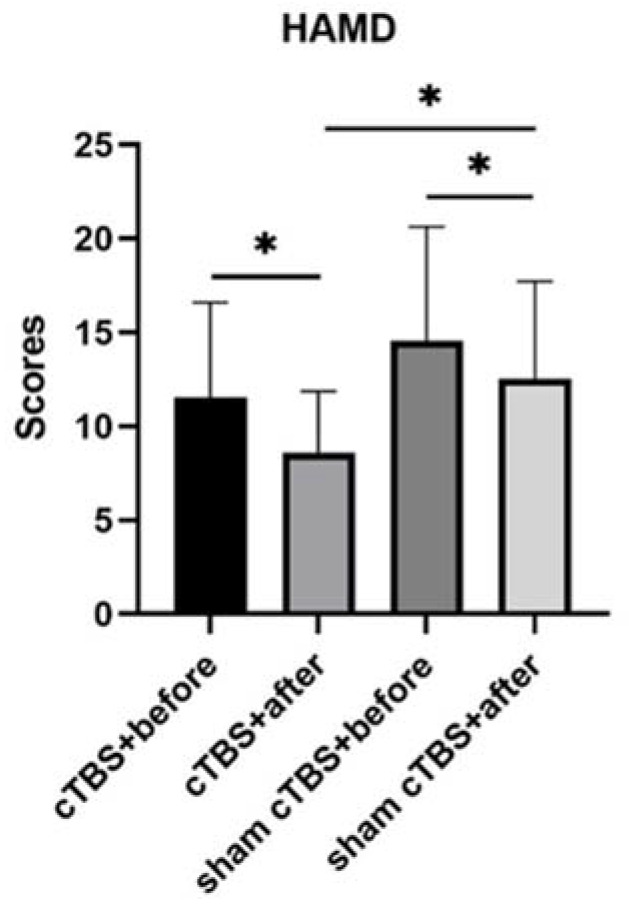
The HAMD scores between the two groups. ^∗^ *p* < 0.05 indicated a statistical significance. HAMD, Hamilton depression; cTBS, continuous theta burst stimulation.

The HAMD scores in both groups improved significantly after treatment compared to before (*p* < 0.05, Figure 1). Moreover, the scores in the cTBS group decreased significantly compared to the sham cTBS group (*p* < 0.05, Figure 1).

### 4.2. The Effects of cTBS on Cognitive Function

**Figure 2:**
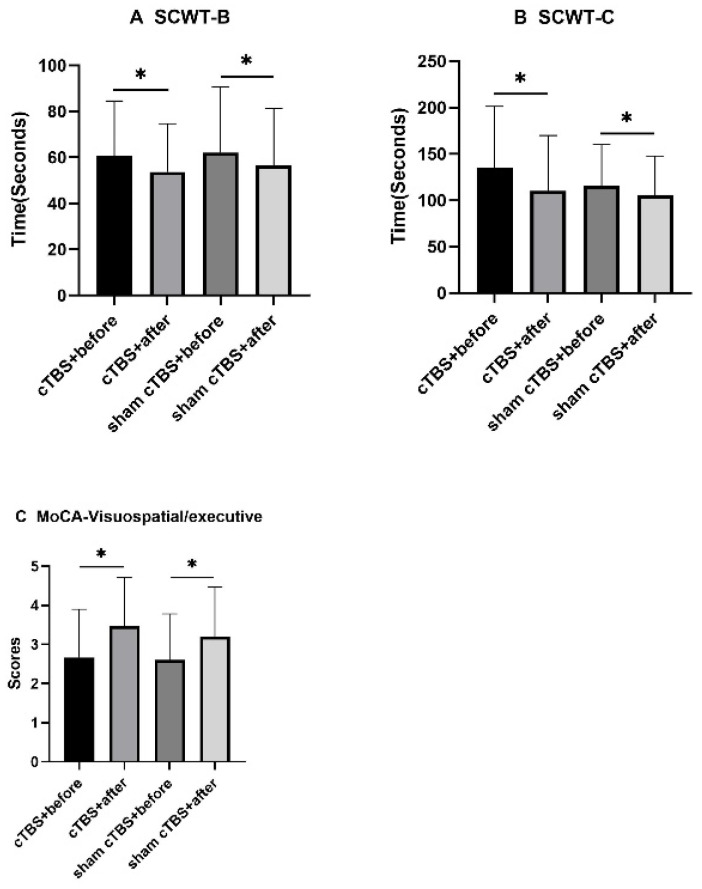
For the MoCA subitem visuospatial/executive scores, the SCWT-B and SCWT-C completion times in the two groups both improved significantly before and after treatment. ^∗^ *p* < 0.05 indicated a statistical significance. MoCA, Montreal Cognitive Assessment; SCWT, Stroop Color-Word Test; cTBS, continuous theta burst stimulation.

The SCWT-B and SCWT-C completion times in both groups decreased significantly before and after treatment (*p* < 0.05, Figure 2A,B), and the MoCA subitem visuospatial/executive scores improved significantly before and after treatment in both groups (*p* < 0.05, Figure 2C).

**Figure 3:**
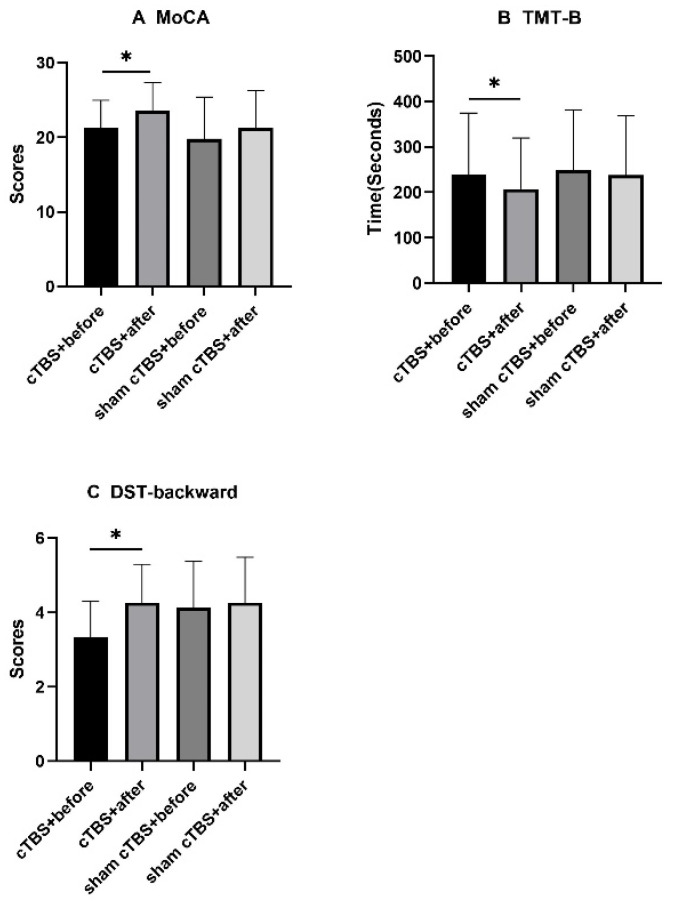
For the MoCA scores, the DST-backward scores and the TMT-B completion times in the cTBS group improved significantly before and after treatment. ^∗^ *p* < 0.05 indicated a statistical significance. MoCA, Montreal Cognitive Assessment; TMT, Trail Marking Test; DST, Digital Span Test; cTBS, continuous theta burst stimulation.

For the MoCA scores, the DST-backward scores improved significantly (*p* < 0.05, Figure 3A,C) and the TMT-B completion times decreased significantly (*p* < 0.05, Figure 3B) in the cTBS group before and after treatment.

**Figure 4:**
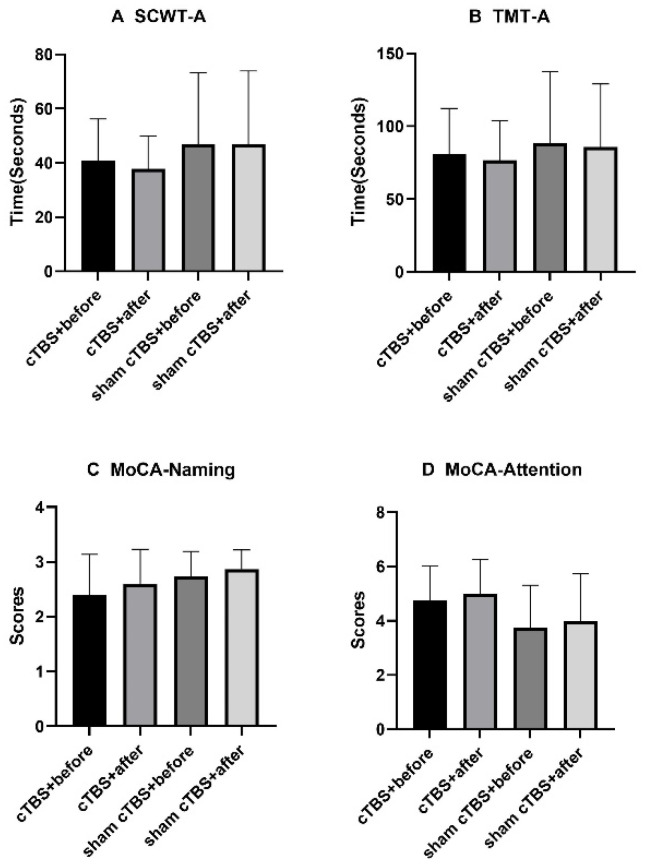
There was no significant difference in SCWT-A, the TMT-A completion times, and the MoCA subitems naming or attention scores either before or after treatment in the two groups or between the two groups. MoCA, Montreal Cognitive Assessment; TMT, Trail Marking Test; SCWT, Stroop Color-Word Test; cTBS, continuous theta burst stimulation.

There was no significant difference in the MoCA subitems naming, attention scores, or the SCWT-A and TMT-A completion times either before or after treatment in the two groups or between the two groups (*p* > 0.05, Figure 4A–D).

**Figure 5:**
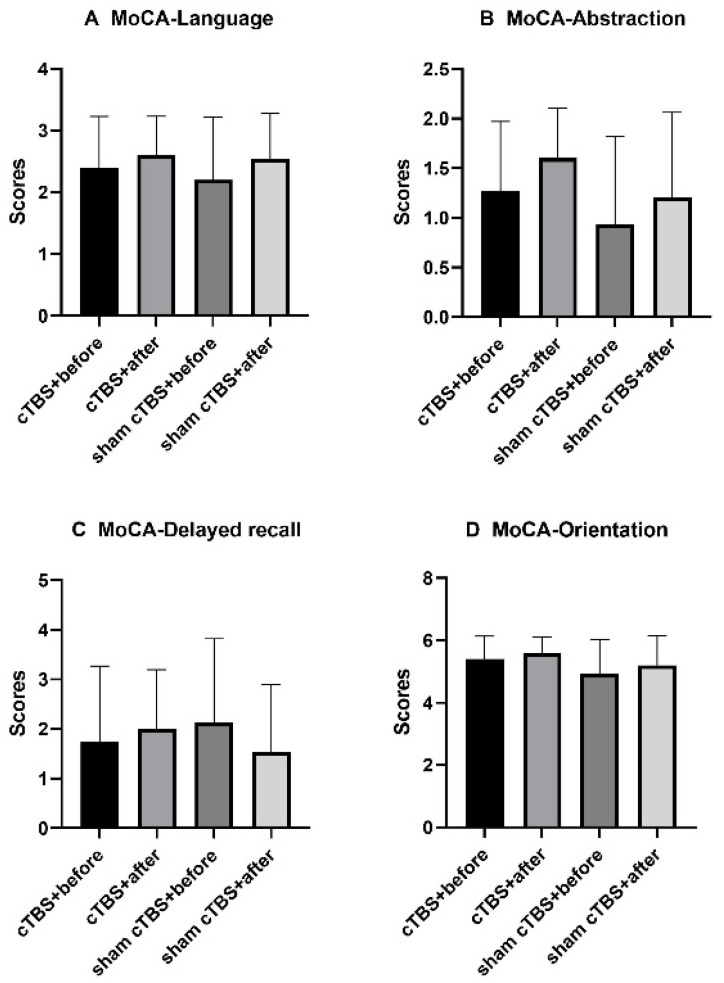
There was no significant difference in the MoCA subitems language, abstraction, delayed recall, or orientation scores either before or after treatment in the two groups or between the two groups. MoCA, Montreal Cognitive Assessment; cTBS, continuous theta burst stimulation.

There was no significant difference in the MoCA subitems language, abstraction, delayed recall, or orientation scores either before or after treatment in the two groups or between the two groups (*p* > 0.05, Figure 5A–D).

## 5. Discussion

In this study, cTBS over the right DLPFC decreased the HAMD scores significantly in patients with CSVD but had no significant improvement or inhibitory effects on cognitive function. As the results showed, the HAMD scores in the cTBS group decreased significantly compared to the control group, and there was no significant difference in the MoCA or DST scores or in the SCWT or TMT completion times between the two groups. For the HAMD scores and the MoCA subitem visuospatial/executive scores, the SCWT-B and SCWT-C completion times in the two groups both improved significantly before and after treatment. For the MoCA scores, the DST-backward scores and the TMT-B completion times in the cTBS group improved significantly before and after treatment. There was no significant difference in SCWT-A, the TMT-A completion times, or the MoCA subitems naming, attention, language, abstraction, delayed recall, or orientation scores either before or after treatment in the two groups or between the two groups.

The dorsolateral prefrontal cortex plays a crucial role in numerous cognitive processes, including attention [29], working memory [30], decision-making [31], inhibitory control [21,32], and depressive symptoms [33]. Non-invasive neuromodulation tools (such as TMS) over the DLPFC could significantly improve cognitive function and depression. As a protocol for TMS, TBS has been shown to have similar or even better effects than TMS for improving depression and cognition [15,34]. TBS is similar to the endogenous theta rhythms of the brain and is thought to modify cortical activity more effectively through the induction of long-term potentiation (LTP)-like and long-term depression (LTD)-like plasticity [10]. However, the choice between iTBS or cTBS, as well as between the right or left DLPFC, is still controversial. More studies have focused on the effects of iTBS over the left DLPFC or right-sided cTBS combined with left-sided iTBS. These previous studies attempted to investigate the cTBS protocol delivered unilaterally to the right DLFPC, but results of improvement, inhibition, or no obvious effect were all observed. Since executive function and attention are often impaired in CSVD patients and are usually accompanied by depression, this study mainly investigated the effect of cTBS over the right DLPFC on cognitive function and the HAMD scores.

Our study showed that the HAMD scores decreased significantly in the cTBS group compared to the sham group. CSVD could lead to depressive symptoms via damage to frontal-subcortical circuits, and a pathological condition was hypothesized to predispose individuals to depression [35]. Songran Yang et al. [35] found that the lesions located in the left thalamus, left putamen, right insular cortex, and right superior longitudinal fascicle were associated with post-stroke depression (PSD). These regions were closely related to the DLPFC. During the treatment of depression using TMS, the left DLPFC function was abnormally weakened, and the right DLPFC function was abnormally enhanced in the patients with depression. Therefore, treatment is usually performed using a stimulus program that excites the left cortex and suppresses the right. Based on the above mechanisms and according to a meta-analysis by Berlim et al. [36], a Level B of evidence (probable antidepressant efficacy) could be proposed for a sequential bilateral left-sided iTBS in addition to a right-sided cTBS protocol applied to the DLPFC in the context of patients with unipolar major depression. On the other hand, unilateral cTBS over the right DLPFC was ineffective for depression score reduction [13]. Our results showed significant effects of cTBS over the right DLPFC in reducing depression scores. We speculated that the previous studies mostly focused on major depression that did not have many vascular risk factors. However, the mechanism of “vascular depression”, a hypothesis proposed by Alexopoulos in 1997 [37], including CSVD-related depression, is not completely the same as major depression. Since CSVD is more common among the elderly, its relevance to depression becomes greater with age. It was postulated that cerebrovascular damage might contribute to depression in advanced age via the involvement of the brain regions involved in mood regulation, notably by damaging the subcortical regions [38]. A recent population-based study revealed that a large white matter hyperintensities (WMH) volume due to CSVD is associated with chronic depression above the age of 60 years [39]. The stimulus protocol of our study may be more effective and suitable for CSVD patients, which can be verified by further expanding the sample size in the future.

According to our results, there was no significant difference between the sham group and the cTBS group in cognition after therapy. The effects of cTBS over the right DLPFC on cognition varied among the different studies. In a systematic review [40], cTBS over the right DLPFC impaired attention, inhibitory control, planning, and goal-directed behavior in decision-making but improved decision-making by reducing impulsivity. Another study found that cTBS over the right DLPFC increased the sensitivity to reinforcers, leading to an avoidance of risky choices and a promotion of advantageous choices [31]. Other studies found that cTBS over the right DLPFC did not improve working memory [30]. Animal experiments showed that the mechanism of cTBS for improving cognitive function might be related to improving the function of the glymphatic system and regulating the polarization of aquaporin-4 [41,42]. In our study, the cognitive function assessment involved the use of the MoCA, SCWT, TMT, and DST. For the MoCA and DST-backward scores, the completion time of TMT-B only in cTBS group improved significantly before and after treatment, suggesting that cTBS over the right DLPFC may improve cognitive function to some extent. However, we did not find any significant difference between the sham and cTBS groups among these assessments. Based on these results, performing cTBS over the right DLPFC did not improve or impair cognitive functions, such as inhibitory control, attention, set-shifting ability, or working memory. These results could have been related to the small sample size, the short session of stimulation, and the lack of regular follow-up evaluations. In addition, the assessment of the MoCA and DST primarily addressed focused attention, which is only one aspect of attention. The other aspects of attention, including selective attention, alternating attention, divided attention, and sustained attention, were not assessed. Some studies found that both an active and passive neuromodulation of the prefrontal cortex [43,44] seemed to be effective on sustained attention. The above evidence might help us explain the absence of certain significant effects of prefrontal cTBS on attention and choose other, more suitable assessment scales for attention.

As mentioned above, there were some limitations in our study. First, the sample size was relatively small. It was difficult to collect patients with CSVD strictly according to the inclusion criteria. The small sample size would affect the generality of the findings. Second, the stimulation protocol was performed once a day for a total of 2 weeks in our study. Maybe the time of stimulation was not enough. Insufficient stimulation may have resulted in unsustainable effects. Third, CSVD is a disease of the whole brain, and we assumed that a circular coil could be more suitable for these patients, which was the direction of our future study. Forth, in this paper, we only completed neuropsychological evaluations, but we also completed functional magnetic resonance imaging (fMRI) before and after treatment for some cases, which could be better analyzed in combination with the neuropsychological evaluation results to some certain extent. However, the number of these cases was much smaller, and we will continue to collect these data in the future. Fifth, we did not have data from regular follow-ups, and thus couldn’t evaluate any possible changes over the course of the disease. Therefore, in our study, the choice of coil, the imaging data, an enlarged sample size, a prolonged treatment session, and a regular follow-up may be necessary in the future to achieve better effects.

In conclusion, our study showed that cTBS over the right DLPFC decreased the HAMD scores significantly in patients with CSVD but had no significant improvement or impairment effects on cognitive function. Additionally, cTBS over the right DLPFC could be used to treat CSVD patients with depression symptoms. In the future, we hope to enlarge the sample size, improve regular follow-ups, and increase the treatment sessions if necessary.

## Figures and Tables

**Table 1:** Patient comparisons.

Group	Age	Sex (n)		**Educational Level**
	**(Years)**	Male	Female	**(Years)**
cTBS	68.93 ± 5.09	11	4	9.87 ± 2.56
Sham cTBS	64.93 ± 6.70	11	4	9.80 ± 3.45

cTBS, continuous theta burst stimulation. *p* > 0.05.

## Data Availability

The data presented in this study are openly available in the “Clinical Trial Management Public Platform”(Number: ChiCTR2100055080). The data is also available upon request from the corresponding authors.

## Abbreviation

CSVD	Cerebral small vessel disease
VCI	Vessel cognitive impairment
rTMS	Repeated transcranial magnetic stimulation
TBS	Theta burst stimulation
cTBS	Continuous theta burst stimulation
iTBS	Intermittent theta burst stimulation
LTP	Long-term potentiation
LTD	Long-term depression
DLPFC	Dorsolateral prefrontal cortex
MoCA	Montreal Cognitive Assessment
SCWT	Stroop Color-Word Test
TMT	Trail Marking Test
DST	Digital Span Test
HAMD	Hamilton depression scale
RMT	Resting motor threshold
MEPs	Motor evoked potentials
M	Median
IQR	Interquartile range
PSD	Post-stroke depression
WMH	White matter hyperintensities
